# Better approach for autoimmune pulmonary alveolar proteinosis treatment: inhaled or subcutaneous granulocyte-macrophage colony-stimulating factor: a meta-analyses

**DOI:** 10.1186/s12931-018-0862-4

**Published:** 2018-08-31

**Authors:** Gaohong Sheng, Peng Chen, Yanqiu Wei, Jiaojiao Chu, Xiaolei Cao, Hui-Lan Zhang

**Affiliations:** 1 Department of Respiratory and Critical Care Medicine, Tongji Hospital of Tongji Medical College, Huazhong University of Science and Technology, No.1095, Jie Fang Road, Han Kou District, Wu Han, 430030 Hu Bei Province China; 2 Division of Cardiology, Departments of Internal Medicine and Genetic Diagnosis Center, Tongji Hospital, Tongji Medical College, Huazhong University of Science and Technology, Wu Han, China; 3 Hubei Key Laboratory of Genetics and Molecular Mechanism of Cardiological Disorders, Wuhan, 430030 China; 4 Division of Respiratory and Critical Care Medicine, the Second Hospital of Huangshi, Huangshi, 435000 China

**Keywords:** Autoimmune pulmonary alveolar proteinosis, Granulocyte-macrophage colony-stimulating factor, Inhaled GM-CSF, Subcutaneous GM-CSF, Meta-analyses

## Abstract

**Background:**

Autoimmune pulmonary alveolar proteinosis (aPAP) is a rare pulmonary disease caused by functional deficiency of granulocyte-macrophage colony-stimulating factor (GM-CSF). GM-CSF therapy in aPAP has been reported effective in some studies. This meta-analyses aimed to evaluate whether GM-CSF therapy, including inhaled and subcutaneous GM-CSF have therapeutic effect in aPAP patients.

**Methods:**

We analyzed 10 studies searched from PubMed, EmBase, Web of Science, Wiley Online Library and Cochrane Collaboration databases to evaluate the pooled effects of GM-CSF treatment in aPAP patients.

**Results:**

Ten observational studies involving 115 aPAP patients were included. The pooled analyses of response rate (81%, *p* < 0.001), relapse rate (22%, *p* = 0.009), PaO_2_ (13.76 mmHg, *p* < 0.001) and P(A-a)O_2_ (19.44 mmHg, *p* < 0.001) showed that GM-CSF treatment was effective on aPAP patients. Further analyses showed that inhaled GM-CSF treatment was more effective than subcutaneous GM-CSF therapy, including a higher response rate (89% vs. 71%, *p* = 0.023), more improvements in PaO_2_ (21.02 mmHg vs. 8.28 mmHg, *p* < 0.001) and P(A-a)O_2_ (19.63 mmHg vs. 9.15 mmHg, *p* < 0.001).

**Conclusions:**

As two routes of exogenous GM-CSF treatment, inhaled and subcutaneous were both proven to have effect on aPAP patients. Furthermore, inhaled GM-CSF therapy showed a higher response rate, more improvements on PaO_2_ and P(A-a)O_2_ than subcutaneous GM-CSF treatment in aPAP patients, suggesting inhaled GM-CSF therapy could have more benefits on aPAP patients. Therefore, GM-CSF therapy, especially inhaled GM-CSF, might be a promising therapeutic option in treating aPAP.

**Electronic supplementary material:**

The online version of this article (10.1186/s12931-018-0862-4) contains supplementary material, which is available to authorized users.

## Background

Pulmonary alveolar proteinosis (PAP), first described by Rosen et al. in 1958 [1], is a rare lung disease characterized by deposition of lipoproteinaceous-rich materials within the alveoli, whose annual prevalence was estimated to be 3.7–6.2 per million [2, 3]. The accumulation of lipoproteinaceous-rich materials was caused by the disability of macrophages to clear alveolar surfactants, which could result in restrictive pulmonary ventilation dysfunction, decreased diffusion capacity, and even could progress to respiratory failure [3–5].

Approximate 90% PAP cases were autoimmune pulmonary alveolar proteinosis (aPAP) characterized by elevated levels of antibodies against granulocyte-macrophage colony-stimulating factor (GM-CSF) in blood and alveoli, and the antibodies could not be detected in health controls [6–9]. In recent years, researchers observed lower ability of alveolar macrophages to clear alveolar surfactants in GM-CSF deficiency mice, and phenotypes similar to aPAP patients histologically were developed in these models [10–12].

Whole lung lavage (WLL) has been used as the standard treatment for aPAP for decades. However, the performance of WLL therapy was limited due to the requirement of anesthesia and invasive procedure [13]. In recent years, some studies reported that inhaled or subcutaneous GM-CSF administration was effective in aPAP patients. Nevertheless, it is debatable whether either or both routes of GM-CSF therapy is effective, and further, which route could achieve better effect.

The purpose of this meta-analyses was to evaluate the effect of GM-CSF therapy including inhaled and subcutaneous routes in aPAP patients.

## Methods

### Search strategy and study selection

**Fig. 1:**
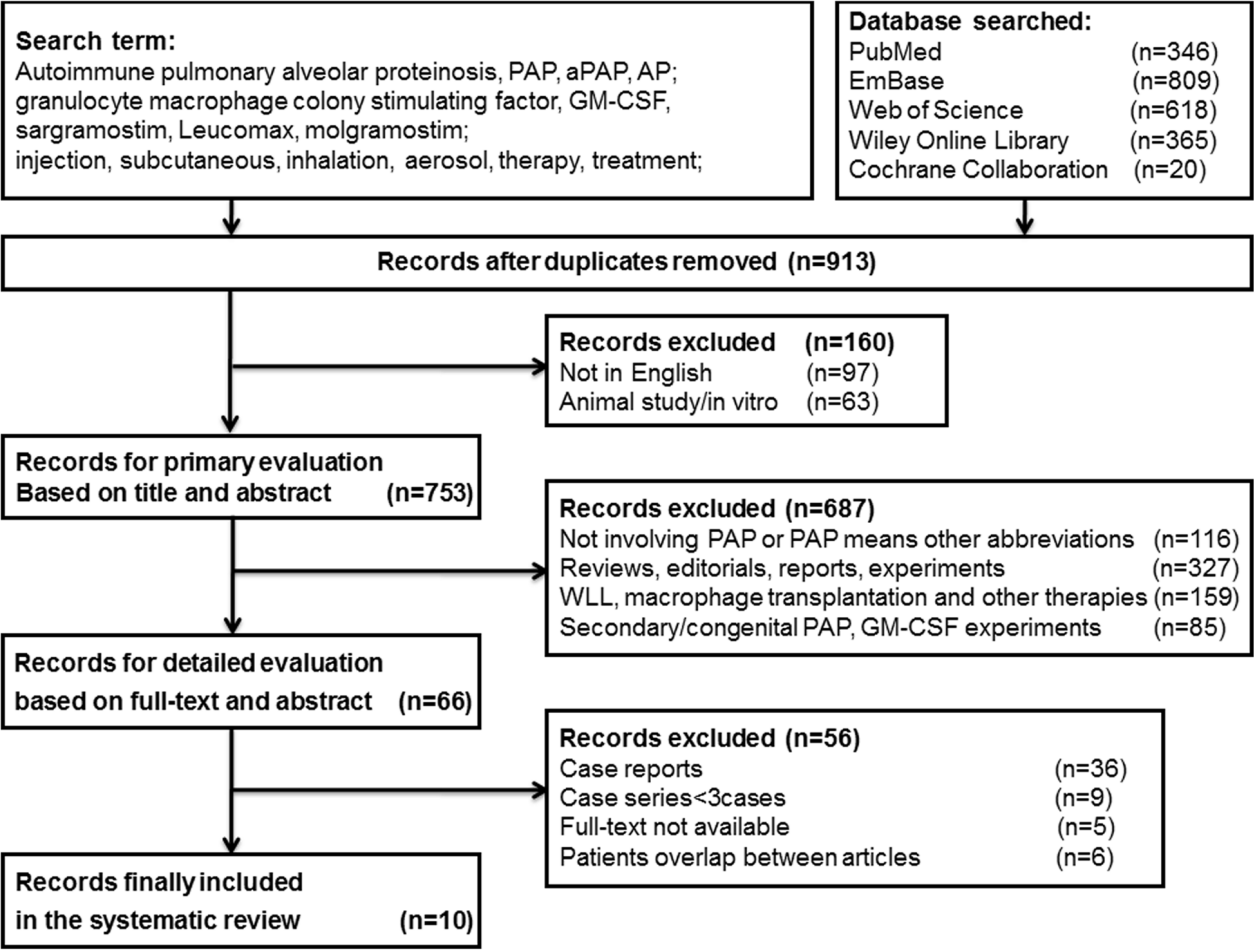
Search flow diagram for studies included in the meta-analyses

The search flow diagram of included studies for this meta-analyses is shown in Fig. 1. We performed our meta-analyses according to the standards set forth by the preferred reporting items for systematic reviews and meta-analyses (PRISMA) statement [14]. We searched PubMed, EmBase, Web of Science, Wiley Online Library and Cochrane Collaboration databases, conference proceedings, trial registers, and other unpublished studies with the terms “Autoimmune pulmonary alveolar proteinosis”, “granulocyte macrophage colony stimulating factor”, “PAP”, “aPAP”, “GM-CSF”, “sargramostim”, “leucomax”, “molgramostim”, “injection”, “subcutaneous”, “inhalation”, “aerosol”, “therapy”, and “treatment”. The included studies were published in English from 1 January 1996 to 30 October 2017, since the first patient receiving GM-CSF therapy was reported in the year of 1996 [15]. The relevant studies in references were also searched manually.

### Inclusion criteria

We included both controlled and uncontrolled studies in English using GM-CSF therapy in aPAP patients aged 18 years or older. The aPAP patients were diagnosed by open lung biopsy or transbronchial lung biopsy, and anti GM-CSF antibody level was examined. The minimum time for GM-CSF therapy should be more than 4 weeks, because there were delays to observe the effect of GM-CSF therapy [16–19].

### Exclusion criteria

Patients diagnosed with secondary or congenital PAP were excluded. In addition, studies not reporting the outcomes of interest, studies with < 3 participants or follow-up time < 3 months were excluded.

A total of 817 studies met the above selection criteria. In the process of screening, 45 case reports were excluded due to low number of participants (*n* < 3). In addition, six studies reporting the same population as other studies were also excluded. And one study was excluded as participants were less than 18-year old. Furthermore, five studies were excluded because their full texts were not available. At last, 10 observational studies satisfying the criteria were included in this meta-analyses.

### Data extraction

Two researchers (Gaohong Sheng and Peng Chen) independently performed literature search and extracted the data from the published studies according to prespecified inclusion/exclusion criteria.

The studies were included or excluded when both researchers made the same judgement for inclusion or exclusion. Disagreements between them were resolved by arbitration of principal investigator (Hui-Lan Zhang). Data on study characteristics, baseline characteristics of patients, and clinical endpoints were extracted and analyzed.

### Endpoint definition

The endpoints of this study were alveolar oxygen partial pressure (PaO_2_), alveolar-arterial oxygen gradient (P(A-a)O_2_), pulmonary function, and disease severity score (DSS) before and after the treatment of GM-CSF. Through synthesizing these parameters, we evaluated therapeutic effects and calculated the response/relapse rate based on the number of response/relapse patients provided in each study. The response criteria were considered as the improvement in PaO_2_, P(A-a)O_2_, pulmonary function, radiology, or symptoms. During the follow-up period, we considered the responders as relapse when these parameters were pejorative.

### Quality assessment

The quality of all included studies was assessed using the Agency for Healthcare Review and Quality (AHRQ) criteria [20]. Each study was assessed by two investigators independently. According to AHRQ score, the included studies were categorized high quality (score > 67), moderate quality (score = 50–66), and low quality (score < 50). Only high and moderate quality studies were eligible for this meta-analyses.

### Data analysis

Data on studies and patients were collected by the two investigators independently. The response and relapse rates were assessed by calculating proportion with 95% confidence intervals (CI) for each study. PaO_2_ and P(A-a)O_2_ were analyzed using weighted mean difference (WMD) with 95% CI. Random effects model was performed to compute the pooled analyses because of the large variety in the GM-CSF therapy. The heterogeneity of the studies was assessed using Cochran’Q statistic, and the magnitude of heterogeneity was estimated using the I^2^ statistic [21]. The impact of small sample studies on the pooled result was evaluated by comparing the results of the fixed to random effects model. Sensitivity analyses were performed to examine stability of pooled outcome. Publication bias was assessed using the funnel plot, Egger’s regression asymmetry test and Begg’s adjusted rank correlation test. In addition, we performed Meta regression analyses with single and multiple covariate based on the age of patients, proportions of men, proportions of smokers, and proportions of patients received WLL.

We also realized that different GM-CSF therapy routes might achieve different effects. Therefore, subgroup analyses was performed according to the routes of GM-CSF therapy. Random effects model was performed in each subgroup to compute the pooled analyses, and differences between the subgroups were analyzed. In addition, we also conducted subgroup analyses according to the proportions of WLL therapy, age of patients, proportions of men and proportions of smokers.

All comparisons were two-tailed, and *p* < 0.05 were recognized as statistical significance. STATA software 14.0 was used to perform statistical analyses.

## Results

### Study characteristics

Ultimately, 10 observational studies were included in this meta-analyses. In these studies, participants in 5 studies received subcutaneous GM-CSF therapy [17, 22–25], and patients in the other 5 studies received inhaled GM-CSF therapy [26–30]. The 10 included studies were published from 2000 to 2016 with follow-up time from 4 months to 10 years. Among them, there were 4 prospective studies and 6 retrospective studies. All the patients in the 10 studies were diagnosed by lung biopsy or bronchoalveolar lavage or computed tomography. More details of the study characteristics were shown in Additional file 1: Table S1. All 10 included studies were eligible to further analyses according to the AHRQ criteria (Additional file 1: Table S2).

### Patient characteristics

A total of 115 patients were included in this meta-analyses, the baseline characteristics of the patients in the included studies were listed in Table 1. The mean age of patients was 46.9 years (95% CI, 45.5–48.4). The mean proportion of men was 60%. On average, 52% patients had the history of smoking, and 57% received WLL therapy combined with GM-CSF. No serious side effects were observed during the course of treatment or follow-up period. And the mean follow-up duration was 3.2 years (95% CI, 2.9–3.5). Data on PaO_2_, P(A-a)O_2_, pulmonary function and the severity of symptoms of the included studies were listed in Additional file 1: Table S3.

**Table 1:** Baseline characteristics of all included patients

Study author/year	No.	Sex, M/F	Age, y	Ever smoking	Anti GM-CSF Ab	GM-CSF dose	GM-CSF duration	WLL	Response	Relapse	Side effect, N
Subcutaneous											
Kavuru et al. [22]	4	4/0	34.3 ± 11.2	3	NA	1-4wk, 250μg/d; 5-8wk, up to 5μg/kg/d; 9^th^wk, up to 7-9μg/kg/d	12 wk	3	3	0	nausea and emisis, 1
Seymour et al. [17]	14	9/5	33 (14–78)	7	12^a^	1-5d 3.0μg/kg/d; 6^th^d, up to 5.0μg/kg/d; if no response, up to 7.5-30 μg/kg/d	12 wk.	10	6	5	neutropenia, 1; first dose effect, 4; local erythema, 6; headache, 1; fever, 1; asymptomatic splenomegaly 1
Venkateshiah et al. [23]	25	18/7	45 (21–57)	13	25	1-4wk, 250μg/d; 5-8wk, up to 5μg/kg/d; 9^th^wk, up to 9μg/kg/d; if no response or suboptimal, up to 9-18μg/kg/d	52 wk	21	12	4	erythema, 18; fatigue, 7; fever, 4; dyspnea, 10; injection-site edema, 12
Khan et al. [25]	4	3/1	40 ± 5.2	NA	NA	5μg/kg/d	12 wk	3	4	0	NA
Hadda et al. [24]	3	0/3	48.3 ± 22	NA	2^a^	3-5 μg/kg/d	6 wk	3	3	0	neutrophilic leukocytosis, 1
Inhaled											
Wylam et al. [26]	12	7/5	42.8 (22–63)	8	6^a^	250μg bid every other week; if no response up to 500μg bid	32 wk	2	11	5	no side effects
Tazawa et al. [27]	39	22/17	56 (46–63)	21	39	high dose: 1-8d, 250μg/d, 9-14d none, six 2-wk cycles; low dose: 1-4d, 125μg/d, 5-14d none, six 2-wk cycles	24 wk	11	24	1	total, 7; fever, 1; respiratory infection,1; otitis media, 1; gastric ulcer, 1; diarrhea, 1; pneumonia, 1; tuberculous lymphadenitis, 1
Papiris et al. [28]	6	1/5	43.8 ± 15.7	2	6	1-4d, 250μg/d, 5-8d none, as long as necessary; if remission, dose down; if relapse, dose up;	14–65 mo	5	6	2	no significant adverse effects
Tazawa et al. [29]	3	1/2	54.7 ± 3.2	2	3	125μg, bid, during alternate weeks	24 wk	1	3	1	no side effects
Ohkouchi et al. [30]	5	4/1	45.8 ± 15.7	4	5	1-8d, 125μg bid, 9-14d, none, six 2-wk cycles; 1-4d, 125μg bid, 5-14d, none, six 2-wk cycles	24 wk	5	5	0	NA

All data are given in median (range) or mean ± SD;

*No* number, *M/F* male/female, *WLL* whole lung lavage, *GM-CSF Ab* granulocyte macrophage colony stimulating factor antibody;

*d* day, *wk* week, *mo* month, *NA* not available; ^a^ the number of detected patients and all were positive

### Response rate

**Fig. 2:**
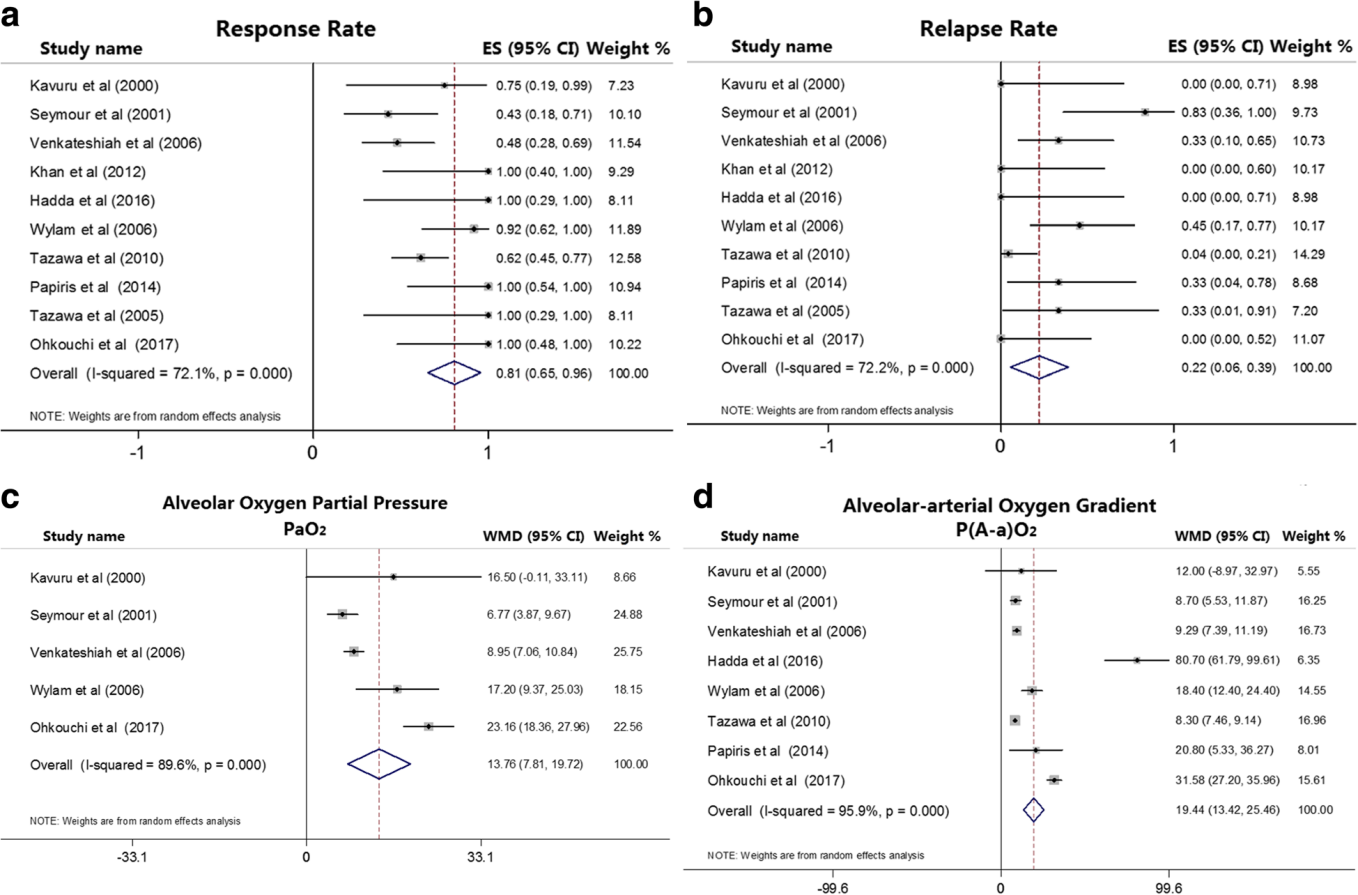
Forest plot shows the pooled outcomes of granulocyte macrophage colony stimulating factor (GM-CSF) therapy in patients with autoimmune pulmonary alveolar proteinosis (aPAP). **a**. Response rate of GM-CSF therapy (81%, 95% CI: 65–96%, *p* < 0.001). **b**. Relapse rate of GM-CSF therapy (22%, 95% CI: 6–39%, *p* = 0.009). **c**. Alveolar oxygen partial pressure (PaO_2_) improved by 13.76 mmHg (95% CI: 7.81–19.72, *p* < 0.001) after GM-CSF therapy. **d**. Alveolar-arterial oxygen gradient (P(A-a)O_2_) decreased by 19.44 mmHg (95% CI: 13.42–25.46, *p* < 0.001) after GM-CSF therapy

In a total of 115 aPAP patients, 77 patients were showed response to GM-CSF therapy. The pooled response rate for all 10 included studies was 81% (95% CI: 65–96%, *p* < 0.001) (Fig. 2a). The degree of heterogeneity was I^2^: 72.1% and *p* < 0.001.The funnel plot (Additional file 1: Figure S1a) showed no publication bias by visual qualitative evaluation, and the quantitative assessments with Begg’s test (*p* = 0.47) (Additional file 1: Figure S1b) and Egger’s test (*p* = 0.68) (Additional file 1: Figure S1c). There were no obvious influences caused by small sample studies (Additional file 1: Figure S2) and each single study through sensitivity analyses (Additional file 1: Figure S1d).

### Relapse rate

In a total of 115 aPAP patients, 18 patients relapsed during GM-CSF therapy or follow-up period. The pooled relapse rate was 22% (95% CI: 6–39%, *p* = 0.009) (Fig. 2b). The degree of heterogeneity was I^2^: 72.2% and *p* < 0.001.The funnel plot (Additional file 1: Figure S3a) showed no visible publication bias by qualitative evaluation, and the quantitative assessments with Begg’s test (*p* = 0.18) (Additional file 1: Figure S3b) and Egger’s test (*p* = 0.17) (Additional file 1: Figure S3c). There were no obvious influences caused by small sample studies (Additional file 1: Figure S4) and each single study through sensitivity analyses (Additional file 1: Figure S3d).

### Alveolar oxygen partial pressure (PaO_2_)

Five studies provided the data of PaO_2_ pre- and post-GM-CSF therapy in total 55 patients. Compared to baseline PaO_2_, the PaO_2_ after GM-CSF therapy was improved by 13.76 mmHg (95% CI: 7.81–19.72, *p* < 0.001) (Fig. 2c). The degree of heterogeneity was I^2^: 89.6% and *p* < 0.001. The funnel plot (Additional file 1: Figure S5a) showed no publication bias by visual qualitative evaluation. And no publication bias was found by Begg’s test (*p* = 0.639) (Additional file 1: Figure S5b) and Egger’s test (*p* = 0.806) (Additional file 1: Figure S5c). No statistical differences were found by sensitivity analyses (Additional file 1: Figure S5d).

### Alveolar-arterial oxygen gradient (P(A-a)O_2_)

The data on P(A-a)O_2_ pre- and post-GM-CSF therapy were available in 8 studies with a total of 103 patients. Through synthetic analyses, one study [24] was obviously different with others, but there was no significant impact of this study on the pooled effect by sensitivity analysis (Additional file 1: Figure S6d). At last, we analyzed the whole 8 studies to evaluate the reduction of P(A-a)O_2_. And the P(A-a)O_2_ was decreased by 19.44 mmHg (95% CI: 13.42–25.46, *p* < 0.001) (Fig. 2d) between pre- and post- GM-CSF therapy. The degree of heterogeneity was I^2^: 95.9% and *p* < 0.001. The funnel plot (Additional file 1: Figure S6a) showed that one study [24] was visually asymmetrical. However, the quantitative assessments showed that no publication bias was found by Begg’s test (*p* = 0.187) (Additional file 1: Figure S6b) and Egger’s test (*p* = 0.266) (Additional file 1: Figure S6c). Thus, we believed there was no publication bias in the pooled analyses.

We also performed analyses on diffusing capacity of the lung for carbon monoxide (DLCO), total lung capacity (TLC), vital capacity (VC), forced expiratory volume in one second (FEV1), forced vital capacity (FVC), DSS, and 6 min’ walk distance (6MWD) (Additional file 1: Figure S8), the details were shown in supplements.

### Inhaled versus subcutaneous GM-CSF

#### Response rate

**Fig. 3:**
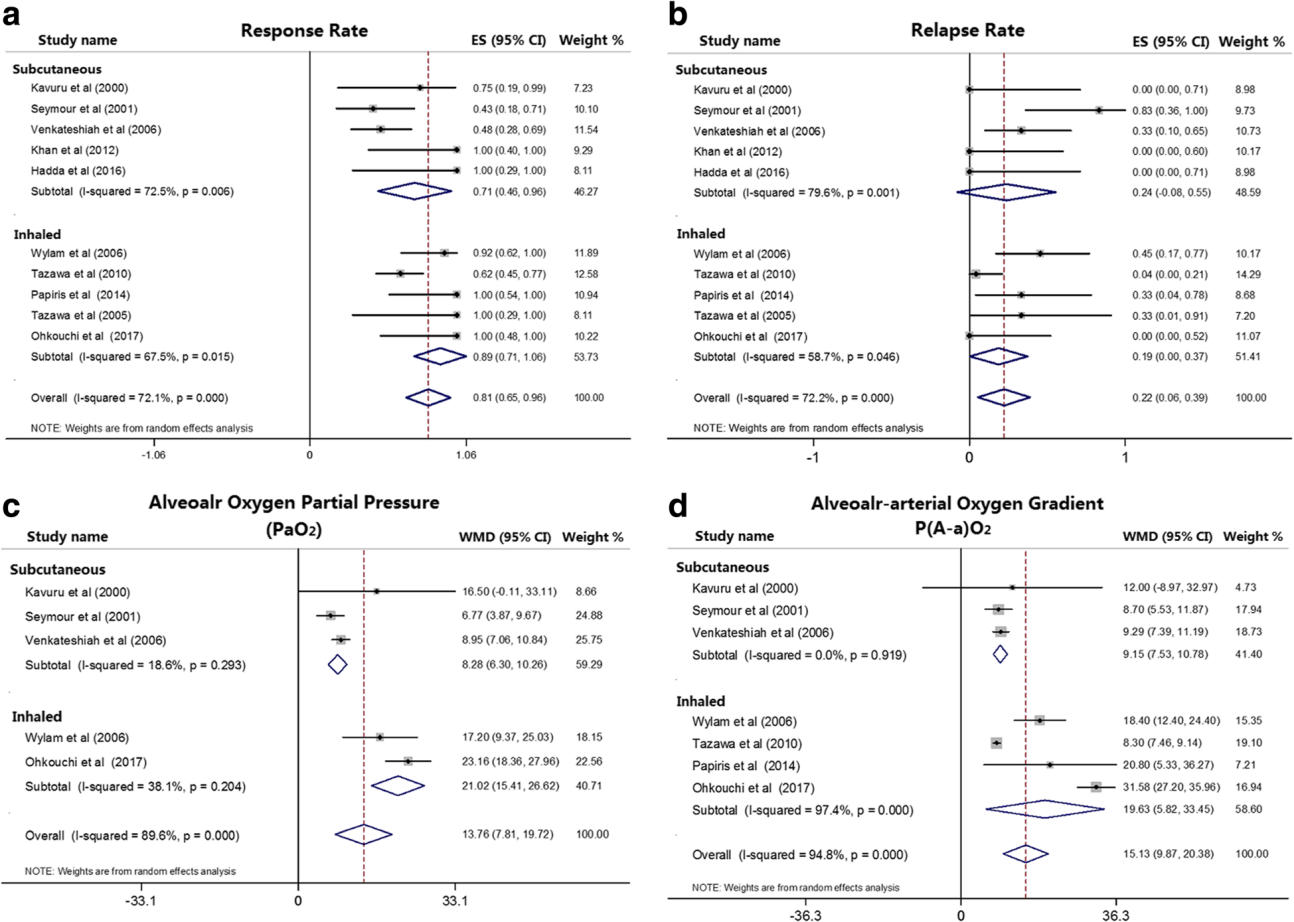
Forest plot shows the pooled outcomes of granulocyte macrophage colony stimulating factor (GM-CSF) therapy in subcutaneous group and inhaled group. **a**. The response rate of GM-CSF therapy in inhaled group (89%, 95% CI: 71–106%, *p* < 0.001) was higher than in subcutaneous group (71%, 95% CI: 46–96%, *p* < 0.001). **b**. The differences in relapse rate were not significant between inhaled group (19%, 95% CI: 0–37%, *p* < 0.05) and subcutaneous group (24%, 95% CI: -8-55%, *p* = 0.14). **c**. Alveolar oxygen partial pressure (PaO_2_) improved more in inhaled group (21.02 mmHg, 95% CI: 15.41–26.62, *p* < 0.001) than in subcutaneous group (8.28 mmHg, 95% CI: 6.30–10.26, *p* < 0.001) after GM-CSF therapy. **d**. Alveolar-arterial oxygen gradient (P(A-a)O_2_) decreased more in inhaled group (19.63 mmHg, 95% CI: 5.82–33.45, *p* = 0.005) than in subcutaneous group (9.15 mmHg, 95% CI: 7.53–10.78, *p* < 0.001) after GM-CSF therapy

The subcutaneous subgroup showed a response rate of 71% (95% CI: 46–96%, *p* < 0.001), while the inhaled subgroup was 89% (95% CI: 71–106%, *p* < 0.001) (Fig. 3a), suggesting both inhaled and subcutaneous GM-CSF therapies were effective for aPAP patients. Furthermore, the differences of response rate between inhaled and subcutaneous subgroups were significant (*p* = 0.023). These data suggested that inhaled GM-CSF was more effective in aPAP patients.

#### Relapse rate

There were 5 studies assigned to subcutaneous subgroup and the pooled relapse rate was 24% (95% CI: -8-55%, *p* = 0.14) (Fig. 3b). The other 5 studies were assigned to inhaled subgroup and the pooled relapse rate was 19% (95% CI: 0–37%, *p* < 0.05) (Fig. 3b). The differences between inhaled and subcutaneous subgroups were not significant (*p* = 0.262).

### Alveolar oxygen partial pressure (PaO_2_)

In total, five studies showed data on PaO_2_ pre- and post-therapy. Significant increase in PaO_2_ was observed in both subcutaneous group (8.28 mmHg, 95% CI: 6.30–10.26, *p* < 0.001) and inhaled group (21.02 mmHg, 95% CI: 15.41–26.62, *p* < 0.001) (Fig. 3c). The difference between inhaled and subcutaneous subgroups was statistically significant (*p* < 0.001).

### Alveolar-arterial oxygen gradient (P(A-a)O_2_)

In the studies of subcutaneous subgroup, sensitivity analyses showed one study [24] had a significant impact on the pooled effects (Additional file 1: Figure S7). Thus, this study was not included in the further analyses. The reductions in (P(A-a)O_2_) were significant in both subcutaneous group (9.15 mmHg, 95% CI: 7.53–10.78, *p* < 0.001) and inhaled group (19.63 mmHg, 95% CI: 5.82–33.45, *p* = 0.005) (Fig. 3d). The mean reduction of P(A-a)O_2_ was larger in inhaled group, and the difference between these two subgroups was statistically significant (*p* < 0.001).

### Meta regression

Results of meta regression analyses suggested effects of GM-CSF therapy were independent of the age of patients, proportions of men, history of smoking, and proportions of combined WLL therapy (*p* > 0.05), respectively.

### Subgroup analyses

We performed subgroup analyses based on the age of patients, and the proportions of men, smokers, anti GM-CSF antibody titer, initial dose and patients combined with WLL therapy. In the studies with more smokers, higher response rate, more improvements in PaO_2_ and P(A-a)O_2_ were found, and the differences between these subgroups were significant. And response rate was significantly higher in the high initial dose subgroup. Relapse rate was found significantly higher in the subgroup with younger age and higher anti GM-CSF antibody titer. The P(A-a)O_2_ improvement was more significant in the subgroup which more patients using WLL therapy. The details were shown in Table 2.

**Table 2:** Subgroup analysis of the effects of GM-CSF therapy on patients with aPAP

Subgroup	Response rate				Relapse rate			
	Studies/patients, n/N	Response rate [95% CI]	*p* value	*p* value for interaction	Studies/patients, n/N	Relapse rate [95% CI]	*p* value	*p* value for interaction
Route of GM-CSF								
Subcutaneous	5/50	0.71 [0.46–0.96]	< 0.001	0.023	5/50	0.24 [−0.08–0.55]	0.14	0.262
Inhaled	5/65	0.89 [0.71–1.06]	< 0.001		5/65	0.19 [0.00–0.37]	0.047	
Age, years								
< 45	5/40	0.83 [0.61–1.04]	< 0.001	0.18	5/40	0.33 [0.01–0.64]	0.04	0.006
≥ 45	5/75	0.79 [0.57–1.01]	< 0.001		5/75	0.10 [−0.03–0.23]	0.13	
Gender, men %								
< 70	6/77	0.81 [0.62–1.01]	< 0.001	0.543	6/77	0.32 [0.05–0.60]	0.02	0.349
≥ 70	4/38	0.80 [0.51–1.09]	< 0.001		4/38	0.09 [−0.08–0.26]	0.293	
Smoker, %								
< 60	4/84	0.63 [0.40–0.86]	< 0.001	0.002	4/84	0.37 [0.01–0.72]	0.041	0.84
≥ 60	4/24	0.93 [0.80–1.07]	< 0.001		4/24	0.19 [−0.06–0.43]	0.136	
Combined therapy with WLL, %								
< 80	6/76	0.77 [0.59–0.96]	< 0.001	0.962	6/76	0.27 [0.00–0.53]	0.047	0.965
≥ 80	4/39	0.86 [0.57–1.15]	< 0.001		4/39	0.16 [−0.03–0.35]	0.098	
Anti GM-CSF antibody titer								
< 40μg/ml	3/50	0.86[0.58–1.14]	< 0.001	0.051	3/50	0.06[−0.06–0.19]	0.313	0.045
≥ 40μg/ml	3/18	0.95[0.80–1.10]	< 0.001		3/18	0.27[−0.02–0.56]	0.066	
Initial dose of GM-CSF								
≤ 250μg/d	7/96	0.74[0.55–0.93]	< 0.001	0.005	7/96	0.25[0.04–0.47]	0.022	0.751
> 250μg/d	3/19	0.95[0.80–1.10]	< 0.001		3/19	0.16[−0.15–0.46]	0.312	
Subgroup	PaO_2_, mmHg				P[A-a]O_2_, mmHg			
	Studies/patients, n/N	WMD [95% CI]	*p* value	*p* value for interaction	Studies/patients, n/N	WMD [95% CI]	*p* value	*p* value for interaction
Route of GM-CSF								
Subcutaneous	3/38	8.28 [6.3–10.26]	< 0.001	< 0.001	3/38	9.15 [7.53–10.78]	< 0.001	< 0.001
Inhaled	2/17	21.02 [15.41–26.62]	< 0.001		4/62	19.63 [5.82–33.45]	0.005	
Age, years								
< 45	3/29	12.11 [3.65–20.57]	0.005	0.614	4/35	14.14 [6.85–21.44]	< 0.001	0.725
≥ 45	2/26	15.88 [1.96–29.80]	0.025		4/68	24.56 [15.41–33.72]	< 0.001	
Gender, men %								
< 70	2/25	11.32 [1.19–21.46]	0.029	0.712	4/70	11.36 [7.27–15.45]	< 0.001	0.186
≥ 70	3/30	16.00 [4.31–27.69]	0.007		3/30	18.23 [−0.14–36.59]	0.052	
Smoker, %								
< 60	1/13	6.77 [3.87–9.67]	< 0.001	< 0.001	3/58	8.53 [6.87–10.18]	< 0.001	< 0.001
≥ 60	4/42	16.27[7.16–25.37]	< 0.001		4/42	18.40 [5.10–31.69]	0.007	
Combined therapy with WLL, %								
< 80	3/29	12.11 [3.65–20.57]	0.005	0.614	4/68	10.64 [6.87–14.4]	< 0.001	0.006
≥ 80	2/26	15.88 [1.96–29.80]	0.025		4/35	33.59 [14.54–52.64]	0.001	
Anti GM-CSF antibody titer								
< 40μg/ml	1/5	23.16(18.36–27.96)	< 0.001	-^*^	3/50	20.11(1.48–38.74)	0.034	0.516
≥ 40μg/ml	1/12	17.20(9.37–25.03)	< 0.001		2/15	48.88(−12.16–109.92)	0.117	
Initial dose of GM-CSF								
≤ 250μg/d	4/43	13.02(6.36–19.67)	< 0.001	0.715	6/88	14.53(8.85–20.20)	< 0.001	0.466
> 250μg/d	1/12	17.20(9.37–25.03)	< 0.001		2/15	48.88(− 12.16–109.92)	0.117	

*GM-CSF* granulocyte macrophage colony stimulating factor, *aPAP* autoimmune pulmonary alveolar proteinosis, *PaO*_*2*_ alveolar oxygen partial pressure, *P[A-a]O*_*2*_ alveolar-arterial oxygen gradient, *WMD* Weighted Mean Difference; ^*^ only one study included in each subgroup, *p* value for interaction not available

## Discussion

This meta-analyses including 10 studies enrolling 115 patients aimed to evaluate whether GM-CSF therapy including inhaled and subcutaneous routes were effective on aPAP patients. The pooled outcomes of response rate, relapse rate, PaO_2_ and P(A-a)O_2_ indicated that GM-CSF therapy was effective on aPAP patients. Moreover, inhaled GM-CSF therapy had a higher response rate (89% vs. 71%, *p* = 0.023), more improvements in PaO_2_ (21.02 mmHg vs. 8.28 mmHg, *p* < 0.001) and P(A-a)O_2_ (19.63 mmHg vs. 9.15 mmHg, *p* < 0.001) than subcutaneous GM-CSF therapy, suggesting that inhaled route was more effective than subcutaneous route in patients with aPAP.

The WLL therapy, whose response rate ranged from 70 to 84%, has been used as standard treatment for aPAP patients for decades [2, 13, 31]. However, compared to the WLL therapy alone, some studies reported that WLL followed by inhaled GM-CSF therapy had better improvements in pulmonary function and radiology [32, 33]. In this meta-analyses, the pooled response rate of GM-CSF therapy (81%) was no less than WLL therapy. The analyses of pulmonary function (including DLCO, TLC, VC, FEV1, FVC), DSS and 6MWD also confirmed the effectiveness of GM-CSF therapy. Furthermore, the subgroup analyses showed no significant differences for response or relapse rate with regard to the proportion of patients using WLL as combination therapy. These results demonstrated that GM-CSF therapy could be recognized as an alternative optimal method rather than a supplement to WLL therapy.

In 2012, one meta-analyses reported that inhaled GM-CSF therapy showed a trend toward higher response rate in aPAP patients comparing with subcutaneous route, suggesting inhaled route might be a better route for GM-CSF therapy [34]. In this meta-analyses, more studies and patients were involved, and the results confirmed a higher response rate in inhaled subgroup with significance (*p* = 0.023). Furthermore, we also compared relapse rate, the values of PaO_2_ and P(A-a)O_2_ pre- and post-therapy in inhaled and subcutaneous subgroups, the results confirmed that aPAP patients would benefit more from inhaled GM-CSF therapy.

Although the differences in relapse rate were not significant between subcutaneous and inhaled subgroups (*p* = 0.262). We found a significant higher relapse rate in the patients younger than 45 years old than that of older than 45 years old (*p* = 0.006) by subgroup analyses (Table 2). And we also observed that the patients were more prone to relapse with higher titer of anti-GMCSF antibodies (*p* = 0.045) (Table 2). As we know, aPAP is an autoimmune disease, the immunologic function might be associated with the relapse rate of aPAP. In 2011, Frasca et al. reported that the humoral and cellular immune responses were both impaired in aged individuals because of age-related defects in T cells and B cells [35]. And Martin et al. also reported that the age-related dysregulation of B cell might result in lacking of magnitude and significant delay in the responses to challenge in older people [36]. Thus, these results suggested that the higher relapse rate with younger age might be associated with more active immune responses in younger patients.

Although the studies declared GM-CSF treatment was well tolerated, side effects still occurred in some patients during treatment and follow-up inevitably [17, 23, 27, 28]. As multiple side effects could occur in one patient and the duration of follow-up varies from studies, we defined the side effect rate as the number of side effect events divided by the patient-years of follow-up. We performed subgroup analyses based on route, initial dose, and relapse rate, the result showed that side effect rate was higher in subcutaneous subgroup than that of inhaled group (*p* < 0.001) (Additional file 1: Table S4). These results suggested that inhaled GM-CSF therapy, as a local treatment route, would achieve better efficacy with less side effects.

GM-CSF inhaled therapy, as a topical route, might result in higher concentration of GM-CSF deposition in the alveoli, which could achieve a better effect than subcutaneous injection. The size and dissolution of the granular preparation for inhalation therapy were considered as the focus to achieve the optimal effect. Luisetti et al. reported a high efficient nebulizer and described the physical properties, lung deposition modeling, and bioactivity of recombinant GM-CSF [37]. Thus, nebulizer was important for the effect of inhaled route.

Up to now, no consensus have been reached on the treatment of aPAP patients. Each medical institution had their own experiences and applicative treatments on aPAP patients. Only when WLL and GM-CSF therapies were invalid, would other therapies such as rituximab [38], plasmapheresis [39–41], gene therapy [42, 43], pulmonary macrophage transplantation therapy [44–46], and lung transplantation [47] be considered as alternative or adjuvant treatments in some aPAP cases. Although no definite guidelines of GM-CSF therapy were published, GM-CSF therapy, especially inhaled GM-CSF, might be a promising therapeutic option in treating aPAP.

### Study limitations

Drawbacks of this study were the differences in baseline characteristics among included studies, containing age, gender, disease severity, treatment dose and duration etc. Second, aPAP is a rare disease with low prevalence, most researches of this disease were studies with small sample. More large-scale samples and long-term follow-up studies are needed in the future. Third, all the studies included were observational studies, 3 abstracts of randomized controlled trials (RCTs) were found, however, full texts of these RCTs were not available.

## Conclusions

Pooled analyses of available studies suggests that treatment of GM-CSF were effective on aPAP patients with response rate (81%). However, further analyses demonstrated that inhaled GM-CSF therapy showed a higher response rate (89% vs. 71%), more improvements of PaO_2_ and P(A-a)O_2_ than subcutaneous route. These results provided a convincing evidence that GM-CSF therapy might be a promising therapeutic option in treating aPAP, furthermore, inhaled GM-CSF would have more benefits on aPAP patients than subcutaneous route.

## Additional file

Additional file 1: **Table S1.** Basic characteristics of all included studies. **Table S2.** Modified AHRQ Quality Assessment Criteria for Observational Studies. **Table S3.** The endpoints of included studies before and after GM-CSF therapy. **Table S4.** Subgroup analysis of side effects with GM-CSF therapy on patients with aPAP. **Figure S1.** (a). Funnel plot of response rate. (b). Begg’s funnel plot of response rate. (c). Egger’s publication bias plot of response rate. (d). Sensitive analysis of response rate. **Figure S2.** The similarity between random and fixed effect models shows no significant influence on the pooled effect of response rate. **Figure S3.** (a). Funnel plot of relapse rate. (b). Begg’s funnel plot of relapse rate. (c). Egger’s publication bias plot of relapse rate. (d). Sensitive analysis of response rate. **Figure S4.** The similarity between random and fixed effect models shows no significant influence on the pooled effect of relapse rate. **Figure S5.** (a). Funnel plot of PaO2. (b). Begg’s funnel plot of PaO2. (c). Egger’s publication bias plot of PaO2. (d). Sensitive analysis of PaO2. **Figure S6.** (a). Funnel plot of P(A-a)O2. (b). Begg’s funnel plot of P(A-a)O2. (c). Egger’s publication bias plot of P(A-a)O2. (d). Sensitive analysis of P(A-a)O2. **Figure S7.** Sensitive analyses of P(A-a)O2 in subcutaneous group shows one study was significantly different with the others. Figure S8. (a). DLCO improved by 8.49% after GM-CSF therapy. (b). TLC improved by 13.04% after GM-CSF therapy. (c). VC improved by 4.83% after GM-CSF therapy. (d). FEV1 improved by 12.58% after GM-CSF therapy. (e). FVC improved by 8.45% after GM-CSF therapy. (f). DSS improved by 2.1 after GM-CSF therapy. (g). 6MWD improved by 51.96 after GM-CSF therapy.

## Abbreviations

6MWD: 6 min’ walk distance
AHRQ: Agency for healthcare review and quality
aPAP: Autoimmune pulmonary alveolar proteinosis
CI: Confidence interval
DLCO: Diffusing capacity of the lung for carbon monoxide
DSS: Disease severity score
FEV1: Forced expiratory volume in one second
FVC: Forced vital capacity
GM-CSF: Granulocyte-macrophage colony-stimulating factor
P(A-a)O_2_: Alveolar-arterial oxygen gradient
PaO_2_: Alveolar oxygen partial pressure
PAP: Pulmonary alveolar proteinosis
RCTs: Randomized controlled trials
TLC: Total lung capacity
VC: Vital capacity
WLL: Whole lung lavage
