# Supplementary material for: Better approach for autoimmune pulmonary alveolar proteinosis treatment: inhaled or subcutaneous granulocyte-macrophage colony-stimulating factor: a meta-analyses
Source: Respir Res. 2018 Aug 31;19:163. doi: 10.1186/s12931-018-0862-4 (PMC6117963; doi:10.1186/s12931-018-0862-4)
Supplement: Supplementary file 1 — Table S1. Basic characteristics of all included studies. Table S2. Modified AHRQ Quality Assessment Criteria for Observational Studies. Table S3. The endpoints of included studies before and after GM-CSF therapy. Table S4. Subgroup analysis of side effects with GM-CSF therapy on patients with aPAP. Figure S1. (a). Funnel plot of response rate. (b). Begg’s funnel plot of response rate. (c). Egger’s publication bias plot of response rate. (d). Sensitive analysis of response rate. Figure S2. The similarity between random and fixed effect models shows no significant influence on the pooled effect of response rate. Figure S3. (a). Funnel plot of relapse rate. (b). Begg’s funnel plot of relapse rate. (c). Egger’s publication bias plot of relapse rate. (d). Sensitive analysis of response rate. Figure S4. The similarity between random and fixed effect models shows no significant influence on the pooled effect of relapse rate. Figure S5. (a). Funnel plot of PaO2. (b). Begg’s funnel plot of PaO2. (c). Egger’s publication bias plot of PaO2. (d). Sensitive analysis of PaO2. Figure S6. (a). Funnel plot of P(A-a)O2. (b). Begg’s funnel plot of P(A-a)O2. (c). Egger’s publication bias plot of P(A-a)O2. (d). Sensitive analysis of P(A-a)O2. Figure S7. Sensitive analyses of P(A-a)O2 in subcutaneous group shows one study was significantly different with the others. Figure S8. (a). DLCO improved by 8.49% after GM-CSF therapy. (b). TLC improved by 13.04% after GM-CSF therapy. (c). VC improved by 4.83% after GM-CSF therapy. (d). FEV1 improved by 12.58% after GM-CSF therapy. (e). FVC improved by 8.45% after GM-CSF therapy. (f). DSS improved by 2.1 after GM-CSF therapy. (g). 6MWD improved by 51.96 after GM-CSF therapy. [file 12931_2018_862_MOESM1_ESM.doc]

**Diffusing capacity of the lung for carbon monoxide (DLCO)**

There were 4 studies showing the data of DLCO pre and post GM-CSF for 63 patients. The DLCO was improved by 8.49% (95%CI: 1.56-15.41, P=0.016) (**Additional file 1: Figure S8a**) at the end of therapy. The degree of heterogeneity was I^2^: 79.8% and P=0.002.

**Total lung capacity (TLC)**

There were only 2 studies containing the TLC values and the overall effect of TLC was improved by 13.04% (95%CI: 4.48-21.59, P=0.003) (**Additional file 1: Figure S8b**). The degree of heterogeneity was I^2^: 0.0% and P=0.69.

**Vital capacity (VC)**

The overall effect of VC for 2 studies was improved by 4.83% (95%CI: 1.86-7.8, P=0.001) (**Additional file 1: Figure S8c**). The degree of heterogeneity was I^2^: 50.5% and P=0.16.

**Forced expiratory volume in one second (FEV1)**

The overall effect of FEV1 for 2 studies was improved by 12.58% (95%CI: 2.05-23.12, P=0.019) (**Additional file 1: Figure S8d**). The degree of heterogeneity was I^2^: 0.0% and P=0.56.

**Forced vital capacity (FVC)**

The data of FVC was available in 3 studies and a significant improvement was observed by 8.45% (95%CI: 0.003-16.9, P=0.05) (**Additional file 1: Figure S8e**). The degree of heterogeneity was I^2^: 58.8% and P=0.09.

**Disease severity score (DSS)**

There were 2 studies providing the data of DSS. The overall effect of DSS was improved by 2.1 (95%CI: 1.76-2.44, P<0.001) (**Additional file 1: Figure S8f**). The degree of heterogeneity was I^2^: 0.0% and P=0.56.

**6 minutes’ walk distance (6MWD)**

We extracted the 6MWD values from 2 studies. And the pooled effect of 6MWD was improved by 51.96 (95%CI: 37.09-66.83, P<0.001) (**Additional file 1: Figure S8g**). The degree of heterogeneity was I^2^: 0.0% and P=0.47.

**Table S1 Basic characteristics of all included studies.**

| **Study/year** | **Type of study** | **Diagnosis, N** | **Inclusion criteria** | **Exclusion criteria** | **Response criteria** | **Follow-up** | **Type of GM-CSF** |
| --- | --- | --- | --- | --- | --- | --- | --- |
| **Subcutaneous** |  |  |  |  |  |  |  |
| Kavuru  et al. 2000 | prospective;  observational | OLB, 4 | adult; primary iPAP;  certain symptoms;  diffuse infiltrates on CXR;  O_2_ uptake at rest | secondary PAP;  increased risk of side-effects;  renal, cardiac, or liver disease | NA | 20 wk | yeast-derived rhGM-CSF,  Sargramostim,  Immunex Corporation, Seattle |
| Seymour  et al. 2001 | prospective;  observational | pathologic review,14 | acquired PAP;  certain symptoms;  no active pulmonary infection | secondary PAP;  exposed to cytotoxic agents;  corticosteroids or cytokines usage | complete response:  recover in PaO_2_, CT,  and spirometry;  partial response:  ≥50% improvement in CXR, P(A-a) O_2_, and DLCO | 16 (10-45) mo | bacterially-synthesized  rhGM–CSF,  Leucomax, Schering-Plough,  Baulkham Hills, Australia;  yeast-derived rhGM-CSF;  Leukine, Immunex, Seattle |
| Venkateshiah  et al. 2006 | prospective;  observational | OLB, 17  TBLB, 8 | adult; primary iPAP;  certain symptoms;  PaO_2_ >55mmHg on O_2_ uptake ≤6L/min at rest;  diffuse infiltrates on CXR | secondary PAP;  increased risk of side-effects;  renal, cardiac, or liver disease;  PaO_2_ ≤55mmHg on O_2_ >6L/min;  need hospitalization and WLL;  active respiratory infection;  pregnant and lactating women | primary end point:  P(A-a)O_2_ down ≥10mmHg;  secondary end point:  improvement in symptoms, quality of life;  tolerability of GM-CSF;  response sustainability | 39 ± 17.3 mo | yeast-derived rhGM-CSF, Leukine, formerly Immunex Corporation and now  Berlex,  Seattle |
| Khan  et al. 2012 | retrospective | OLB, 3;  TBLB, 1 | certain symptoms;  diagnosis with biopsy | NA | improvement in  symptoms, radiology and  pulmonary function | 2 (0.5-3) y | NA |
| Hadda  et al. 2016 | retrospective;  review of cases | TBLB, 2;  BAL and others, 1 | primary PAP | secondary PAP | improvement in  symptoms, radiology and  pulmonary function | 6 (4-60) mo | NA |
| **Inhaled** |  |  |  |  |  |  |  |
| Wylam  et al. 2006 | retrospective;  review of cases | OLB, 7;  TBLB, 5 | primary PAP | NA | PaO_2_ up ≥10mmHg;  P(A-a)O_2_ down ≥12mmHg;  DLCO up ≥12%;  FVC up ≥7%; | 30.5 (3-68) mo | Leukine,  immunex,  Seattle |
| Tazawa  et al. 2010 | prospective;  phase II trial;  self-controlled | OLB, 5;  TBLB, 13;  BAL, 50;  GM-CSF Ab, 50 | age 20-80y;  lung biopsy or cytology findings;  PaO_2_ <75mmHg;  GM-CSF Ab >3ug/mL | WLL therapy within 6 mo;  GM-CSF or cytokines therapy already;  leukocytosis >12,000/uL;  fever ≥38℃;  hematologic malignancy;  primary or metastatic lung cancer;  serve edema or asthma;  congestive heart failure;  angina; bleeding diathesis;  or other clinical situation as judged by the investigator | primary endpoint:  P(A-a)O_2_ down ≥10mmHg after low-dose therapy compared to that before high-dose;  secondary endpoint:  improvement in  pulmonary function,  serum biomarkers of PAP;  safety of GM-CSF; | 52 mo | rhGM-CSF;  sargramostim, Leukine,  lyophilized formulation,  Berlex,  Seattle |
| Papiris  et al. 2014 | retrospective;  review of cases | Biopsy or BAL;  GM-CSF Ab | NA | NA | absence of symptoms;  O_2_ desaturation <4% at  the 6MWT;  significant radiographic  reduction of the infiltrates | 65 mo | Leukine,  Sargramostim, licensed to  Genzyme Corporation,  SanofiAventis,  Cambridge |
| Tazawa  et al. 2005 | retrospective;  review of cases | BAL,TBLB,  and GM-CSF Ab | idiopathic PAP | NA | P(A-a)O_2_ down ≥10mmHg;  improvement in  symptoms, radiology,  and pulmonary function | NA | Leucomax,  Novartis AG, Basel, Switzerland |
| Ohkouchi  et al. 2017 | retrospective;  review of cases | BAL, chest CT,  and GM-CSF Ab | NA | NA | P(A-a)O_2_ down ≥10mmHg;  improvement in  symptoms, radiology,  and pulmonary function | 10 y | rhGM-CSF,  sargramostim, Leukine,  lyophilized formulation, Berlex, Seattle |

All data are given in median (range) or mean (SD);

NA, not available; iPAP, idiopathic pulmonary alveolar proteinosis; CXR, chest radiograph; OLB, open lung biopsy; TBLB, transbronchial lung biopsy; O_2_,oxygen; BAL, bronchoalveolar lavage; GM-CSF, granulocyte macrophage colony stimulating factor; PaO_2_, alveolar oxygen partial pressure; P(A-a)O_2_, alveolar-arterial oxygen gradient; Ab, antibody; wk, week; mo, month; y, year;

DLCO, diffusing capacity of the lung for carbon monoxide; PR, partial response; rhGM-CSF, recombinant human granulocyte macrophage colony stimulating factor.

**Table S2 Modified AHRQ Quality Assessment Criteria for Observational Studies.**

| **Criteria** | **Score** | **Kavuru**  **et al.**  **2000** | **Seymour**  **et al.**  **2001** | **Venkateshiah**  **et al.**  **2006** | **Tazawa**  **et al. 2010** | **Hadda**  **et al. 2016** | **Wylam**  **et al. 2006** | **Khan**  **et al. 2012** | **Papiris**  **et al. 2014** | **Tazawa**  **et al. 2005** | **Ohkouchi**  **et al.**  **2017** |
| --- | --- | --- | --- | --- | --- | --- | --- | --- | --- | --- | --- |
| ***Study question*** | ***2*** |  |  |  |  |  |  |  |  |  |  |
| Clearly focused and appropriate question | 2 | 2 | 2 | 2 | 2 | 2 | 2 | 2 | 2 | 2 | 2 |
| ***Study population*** | ***8*** |  |  |  |  |  |  |  |  |  |  |
| Description of study population | 5 | 5 | 5 | 5 | 5 | 5 | 5 | 5 | 5 | 5 | 5 |
| Sample size justification | 3 | 0 | 0 | 0 | 0 | 0 | 0 | 0 | 0 | 0 | 0 |
| ***Comparability of subjects for all observational*** | ***22*** |  |  |  |  |  |  |  |  |  |  |
| Specific inclusion/exclusion criteria for all group | 5 | 5 | 5 | 5 | 5 | 3 | 5 | 0 | 5 | 0 | 3 |
| Criteria applied equally to all groups | 3 | 3 | 3 | 3 | 3 | 1 | 3 | 1 | 3 | 3 | 3 |
| Comparability of groups at baseline with regard to disease status and prognostic factor | 3 | 1 | 1 | 1 | 3 | 1 | 3 | 3 | 3 | 1 | 1 |
| Study groups comparable to nonparticipants with  regard to confounding factors | 3 | - | - | - | - | - | - | - | - | - | - |
| Use of concurrent controls | 5 | - | - | - | - | - | - | - | - | - | - |
| Comparability of follow-up among groups at each  assessment | 3 | 3 | 3 | 3 | 3 | 3 | 3 | 3 | 3 | 3 | 3 |
| ***Exposure or intervention*** | ***11*** |  |  |  |  |  |  |  |  |  |  |
| Clear definition of exposure | 5 | 5 | 5 | 5 | 5 | 5 | 5 | 5 | 5 | 5 | 5 |
| Measurement method standard, valid, and reliable | 3 | - | - | - | - | - | - | - | - | - | - |
| Exposure measured equally in all study groups | 3 | - | - | - | - | - | - | - | - | - | - |
| ***Outcome measures*** | ***20*** |  |  |  |  |  |  |  |  |  |  |
| Primary/secondary outcomes clearly defined | 5 | 1 | 1 | 5 | 5 | 0 | 0 | 1 | 1 | 1 | 1 |
| Outcomes assessed blind to exposure or intervention | 5 | - | - | - | - | - | - | - | - | - | - |
| Method of outcome assessment standard, valid,  and reliable | 5 | 5 | 5 | 5 | 5 | 5 | 5 | 5 | 5 | 5 | 5 |
| Length of follow-up adequate for question | 5 | 2 | 3 | 5 | 5 | 3 | 5 | 5 | 5 | 5 | 3 |
| ***Statistical analysis*** | ***19*** |  |  |  |  |  |  |  |  |  |  |
| Statistical tests appropriate | 5 | 4 | 4 | 4 | 4 | 4 | 4 | 5 | 4 | 4 | 4 |
| Multiple comparisons taken into consideration | 3 | - | - | - | - | - | - | - | - | - | - |
| Modeling and multivariate techniques appropriate | 2 | - | - | - | - | - | - | - | - | - | - |
| Power calculation provided | 2 | 0 | 0 | 0 | 0 | 0 | 0 | 0 | 0 | 0 | 0 |
| Assessment of confounding | 5 | 0 | 0 | 0 | 0 | 0 | 0 | 0 | 0 | 0 | 0 |
| Dose-response assessment if appropriate | 2 | 2 | 2 | 2 | 2 | 2 | 2 | 2 | 2 | 2 | 2 |
| ***Results*** | ***8*** |  |  |  |  |  |  |  |  |  |  |
| Measure of effect for outcomes and appropriate  measure of precision | 5 | 5 | 5 | 5 | 4 | 5 | 5 | 5 | 5 | 4 | 5 |
| Adequacy of follow-up for each study group | 3 | 3 | 3 | 3 | 3 | 3 | 3 | 3 | 3 | 3 | 3 |
| **Discussion** | ***5*** |  |  |  |  |  |  |  |  |  |  |
| Conclusions supported by results with possible biases and limitations taken into consideration | 5 | 4 | 5 | 5 | 5 | 5 | 5 | 5 | 5 | 5 | 5 |
| ***Funding or sponsorship*** | ***5*** |  |  |  |  |  |  |  |  |  |  |
| Type and sources of support for study | 5 | 5 | 5 | 5 | 5 | 5 | 0 | 0 | 5 | 5 | 5 |
| **Total** | **100** | **55** | **57** | **63** | **64** | **52** | **50** | **50** | **61** | **53** | **55** |

AHRQ= Agency for Healthcare Review and Quality.

**Table S3 The endpoints of included studies before and after GM-CSF therapy.**

| **Endpoints** |  | **Kavuru**  **et al. 2000** | **Seymour**  **et al. 2001** | **Venkateshiah**  **et al. 2006** | **Hadda**  **et al. 2016** | **Wylam**  **et al. 2006** | **Tazawa**  **et al. 2010** | **Papiris**  **et al. 2014** | **Ohkouchi**  **et al. 2017** |
| --- | --- | --- | --- | --- | --- | --- | --- | --- | --- |
| PaO_2_, mmHg | N  Pre  Post | 4  52.25 ± 8.33  68.75 ± 14.76 | 13  63.23 ± 2.65  70 ± 4.62 | 21  61.81 ± 2.75  70.76 ± 3.45 | NA* | 12  70 ± 7.4  87.2 ± 11.7 | NA | NA | 5  62.24 ± 3.86  85.4 ± 3.89 |
| P(A-a)O_2_, mmHg | N  Pre  Post | 4  47.75 ± 11.26  35.75 ± 18.2 | 13  38.62 ± 3.43  29.92 ± 4.72 | 21  37.81 ± 2.31  28.52 ± 3.79 | 3  118.12 ± 13.7  37.42 ± 9.55 | 12  31.3 ± 7.4  12.9 ± 7.6 | 39  43.6 ± 1.5  35.3 ± 2.1 | 6  49.5 ± 18.9  28.7 ± 4.1 | 5  40.78 ± 4.42  9.2 ± 2.33 |
| DLCO, % | N  Pre  Post | NA | 13  48.62 ± 5.84  48.69 ± 6.47 | NA | NA | 12  65.9 ± 17.9  82.6 ± 16.9 | 33  53.7 ± 2.9  61.4 ± 3.1 | 5  37.6 ± 15.1  61.2 ± 10.9 | NA |
| TLC, % | N  Pre  Post | NA | NA | NA | NA | 7  79.3 ± 11.1  90.6 ± 11.9 | NA | 5  61.6 ± 13  76.4 ± 4.8 | NA |
| VC, % | N  Pre  Post | NA | 13  70.23 ± 5.52  72.69 ± 5.99 | NA | NA | NA | 35  81 ± 2.5  86.8 ± 2.9 | NA | NA |
| FEV1, % | N  Pre  Post | NA | NA | NA | NA | 12  80.4 ± 15.7  91.3 ± 14.1 | NA | 5  65.2 ± 22.4  83.7 ± 12.3 | NA |
| FVC, % | N  Pre  Post | NA | NA | NA | NA | 12  80.4 ± 15.7  93.8 ± 10.2 | 35  80.5 ± 2.5  84.2 ± 3 | 5  66.1 ± 20.3  82.9 ± 9.2 | NA |
| DSS | N  Pre  Post | NA | NA | NA | NA | NA | NA | 6  3.33 ± 0.56  1.33 ± 0.21 | 5  3.4 ± 0.51  1.2 ± 0.2 |
| 6MWD, m | N  Pre  Post | NA | NA | NA | 3  311 ± 56  394.6 ± 52.2 | NA | 22  393 ± 27  444 ± 24 |  |  |

GM-CSF, granulocyte macrophage colony stimulating factor; N, the number of patients received [examination](javascript:void(0);); pre, before GM-CSF therapy; post, after GM-CSF therapy;

PaO_2_, alveolar oxygen partial pressure; P(A-a)O_2_, alveolar-arterial oxygen gradient; DLCO, diffusing capacity of the lung for carbon monoxide; TLC, total lung capacity;

VC, vital capacity; FEV1, forced expiratory volume in one second; FVC, forced vital capacity; WBC, white blood cell; DSS, disease severity score;

6MWD, 6 minutes’ walk distance; NA*, not available in at least one of the two values of pre and post GM-CSF therapy.

**Table S4 Subgroup analysis of side effects with GM-CSF therapy on patients with aPAP.**

| **Subgroup** | **Side-effect rate*** | | | |
| --- | --- | --- | --- | --- |
|  | **Studies/patients, n/N** | **Side-effect rate [95% CI]** | **P value** | **P value for interaction** |
| Route of GM-CSF |  |  |  |  |
| Subcutaneous | 4/46 | 0.65[0.55-0.73] | <0.001 | <0.001 |
| Inhaled | 3/57 | 0.03[0.005-0.06] | <0.001 |  |
| Initial dose of GM-CSF |  |  |  |  |
| ≤250ug/d | 5/88 | 0.32[0.03-0.72] | 0.004 | 0.834 |
| >250ug/d | 2/15 | 0.18[0.12-0.88] | 0.275 |  |
| Relapse rate, % |  |  |  |  |
| <10 | 3/46 | 0.30[0.002-0.81] | 0.032 | 0.764 |
| ≥10 | 4/57 | 0.26[0.001-0.73] | 0.037 |  |

* Side-effect rate=the number of side-effect events divided by patient-years of follow up.


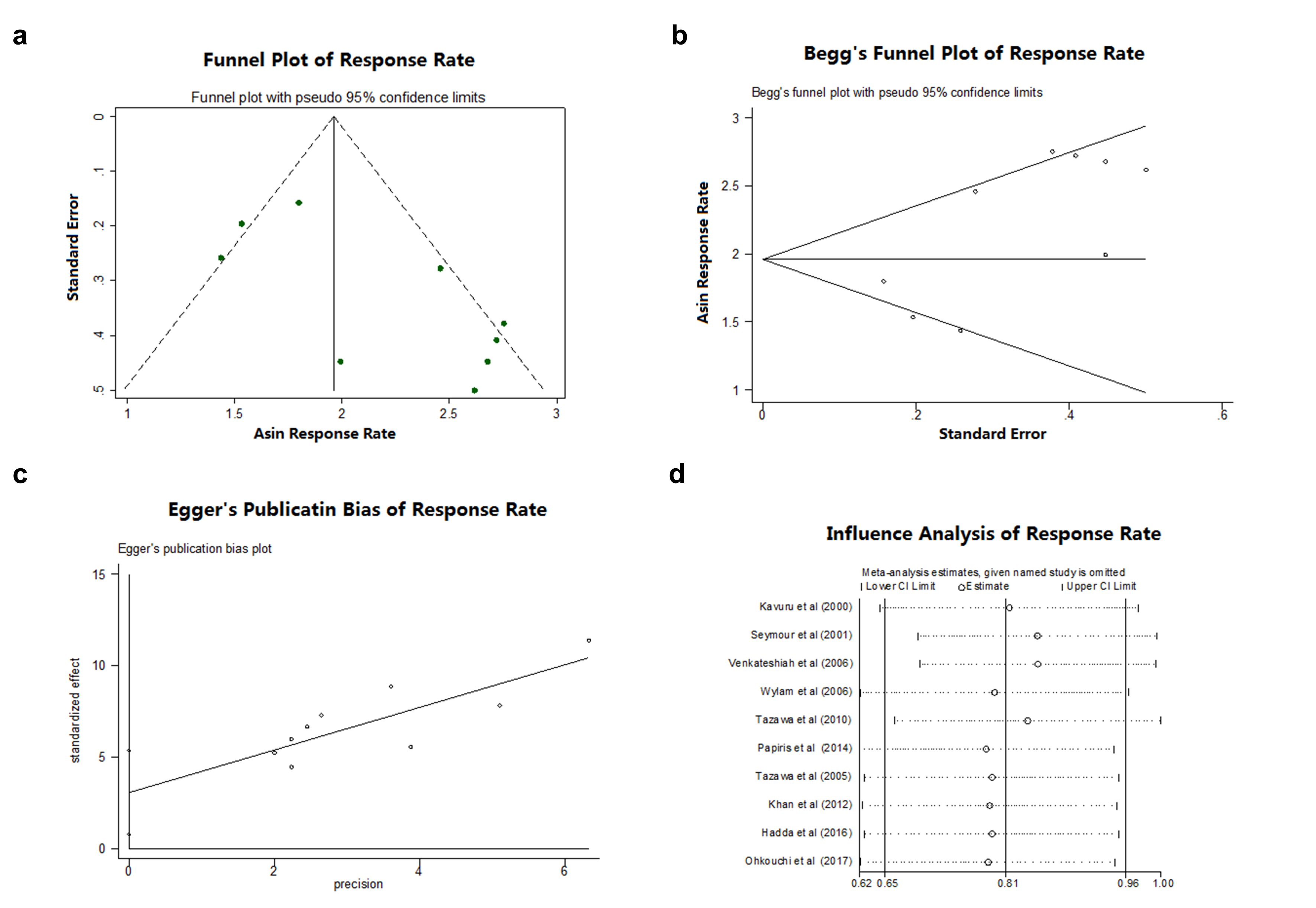


**Figure S1 (a).** Funnel plot showing no publication bias of response rate. **(b).** Begg’s funnel plot showing no publication bias of response rate. **(c).** Egger’s publication bias plot showing no publication bias of response rate. **(d).** Sensitive analysis of response rate shows no significant difference.


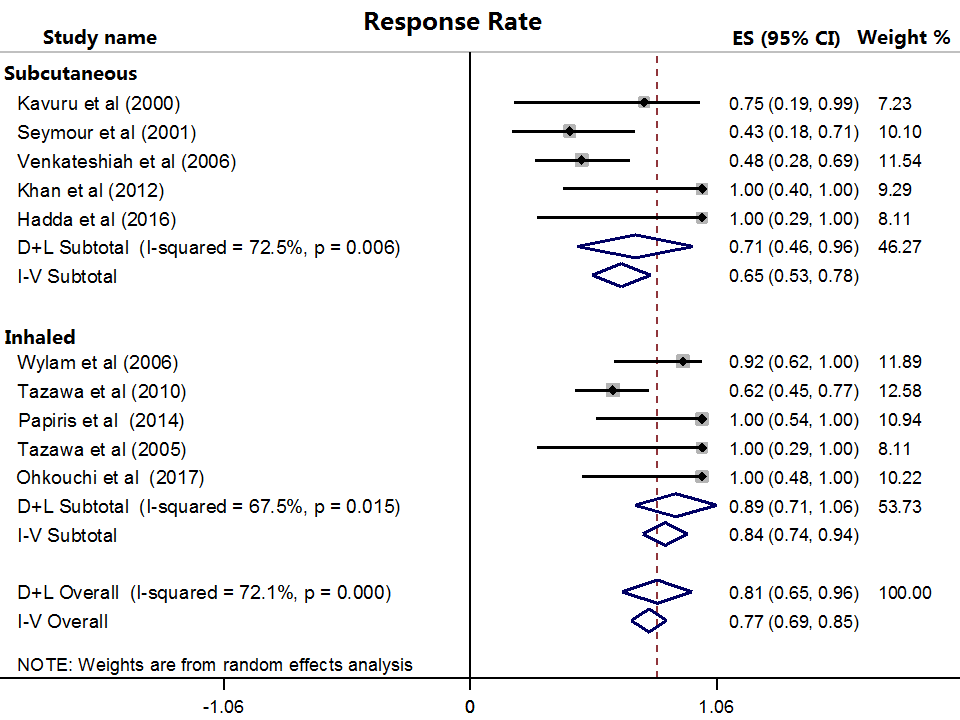


**Figure S2** The similarity between random and fixed effect models shows no significant influence on the pooled effect of response rate.


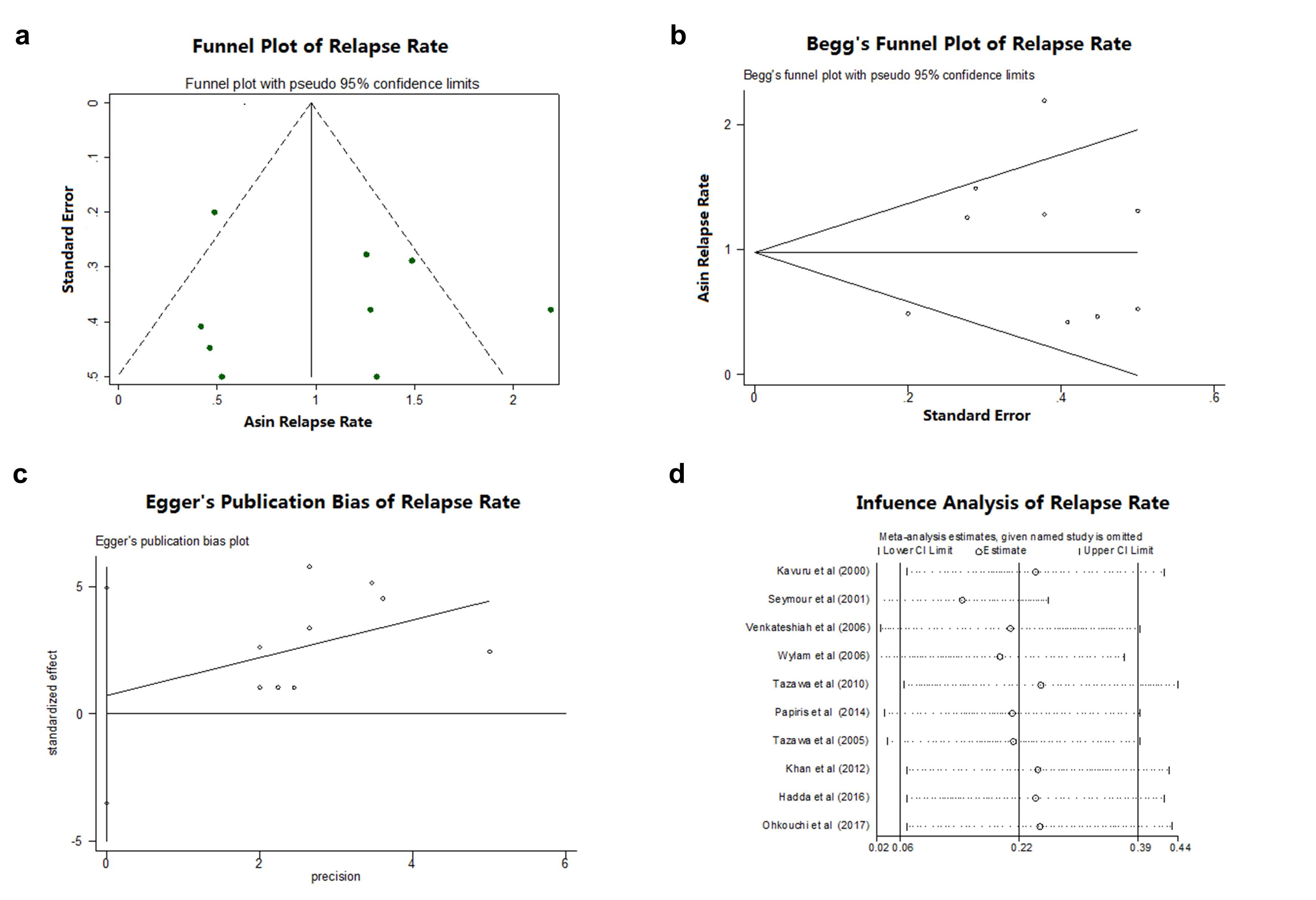


**Figure S3 (a).** Funnel plot showing no publication bias of relapse rate. **(b).** Begg’s funnel plot showing no publication bias of relapse rate. **(c).** Egger’s publication bias plot showing no publication bias of relapse rate. **(d).** Sensitive analysis of response rate shows no significant difference.


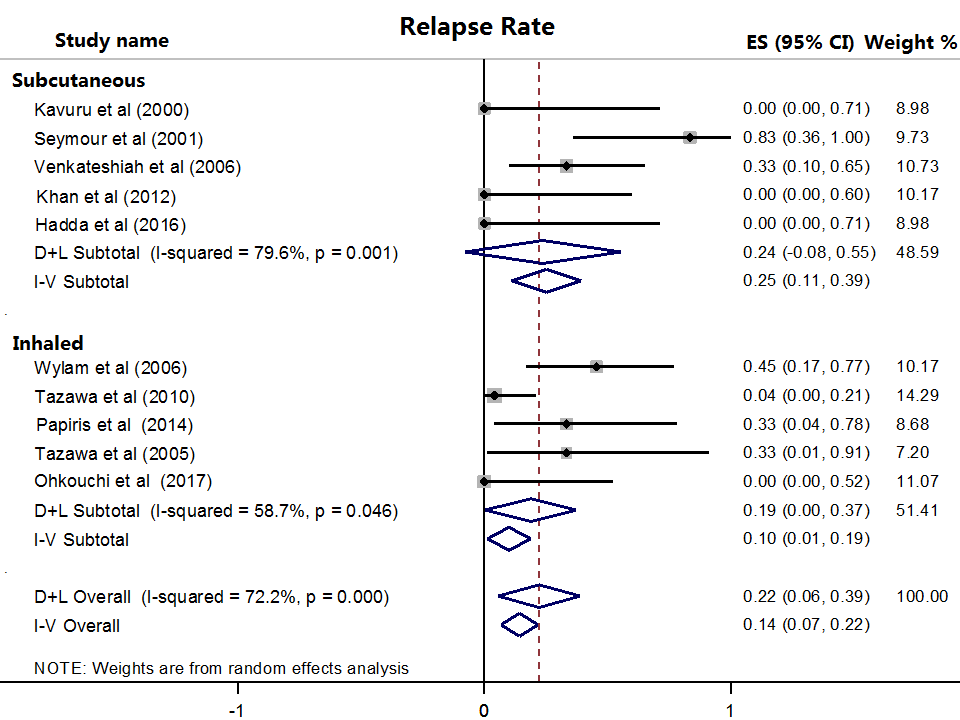


**Figure S4** The similarity between random and fixed effect models shows no significant influence on the pooled effect of relapse rate.


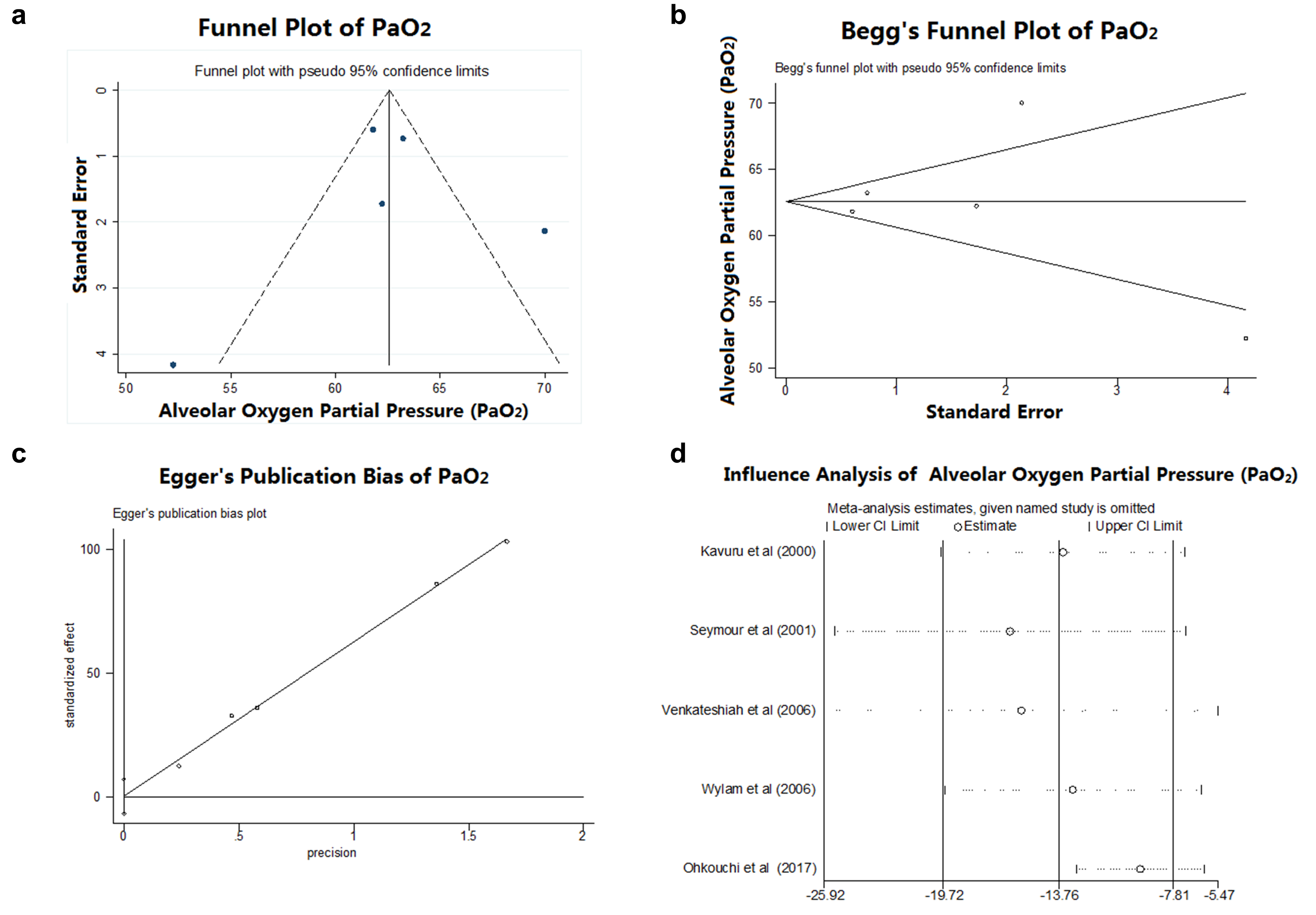


**Figure S5 (a).** Funnel plot showing no publication bias of PaO_2_. **(b).** Begg’s funnel plot showing no publication bias of PaO_2_. **(c).** Egger’s publication bias plot showing no publication bias of PaO_2_. **(d).** Sensitive analysis of PaO_2_ shows no significant difference.


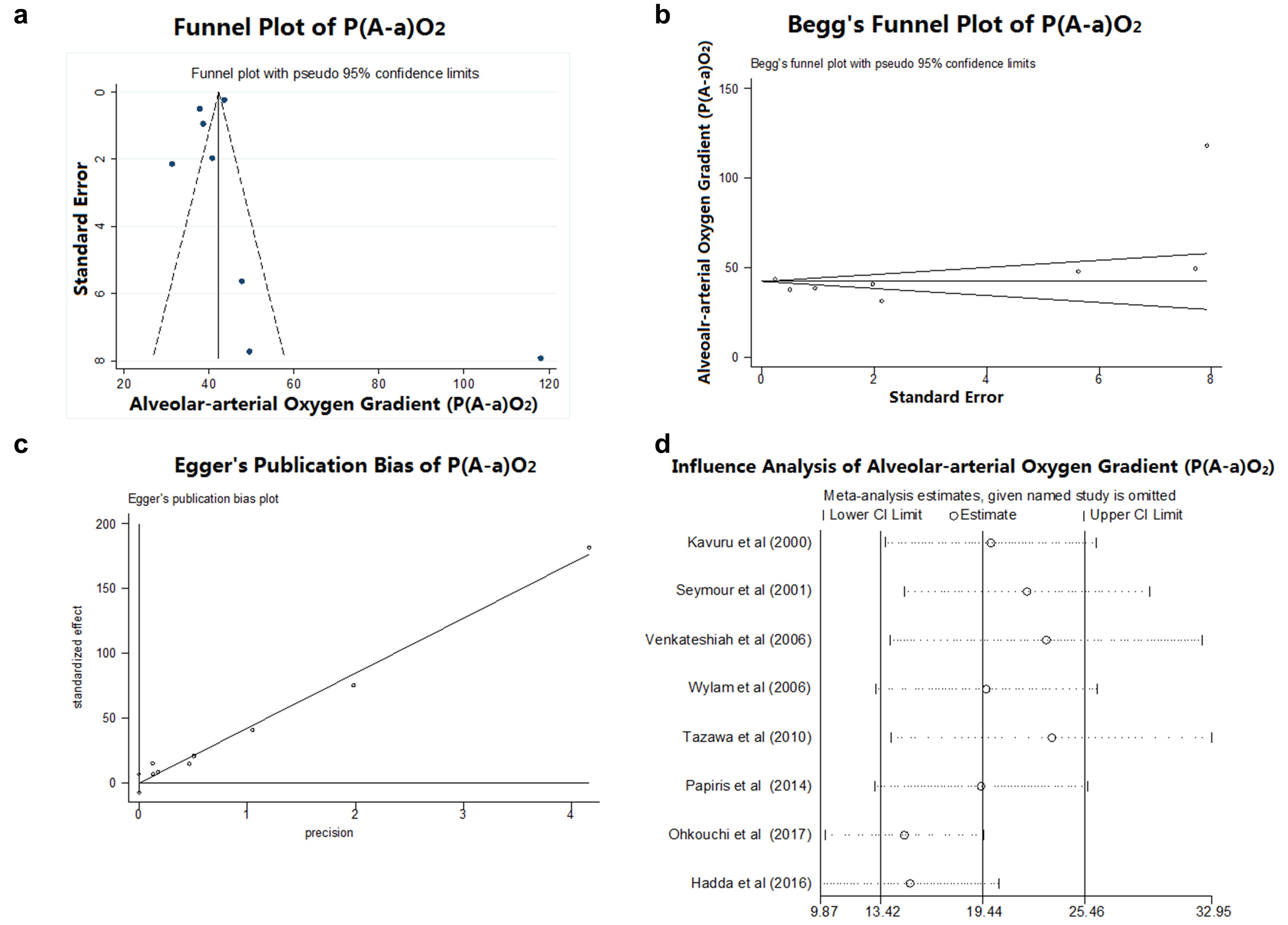


**Figure S6 (a).** Funnel plot of P(A-a)O_2_ showed one study was visually asymmetrical. **(b).** Begg’s funnel plot showing no publication bias of P(A-a)O_2_. **(c).** Egger’s publication bias plot showing no publication bias of P(A-a)O_2_. **(d).** Sensitive analysis of P(A-a)O_2_ shows no significant difference.


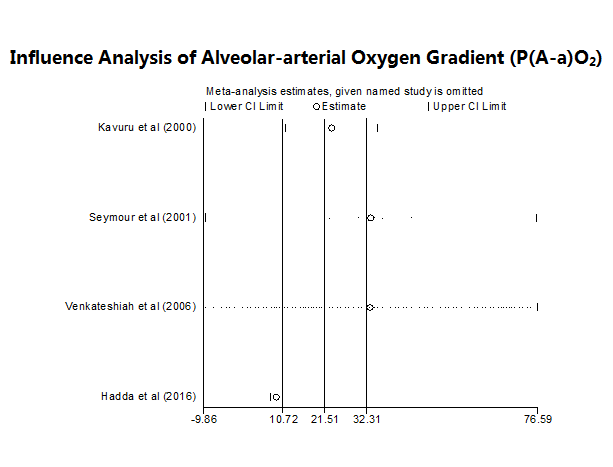


**Figure S7** Sensitive analyses of alveolar-arterial oxygen gradient (P(A-a)O_2_) in subcutaneous group shows one study was significantly different with the others.


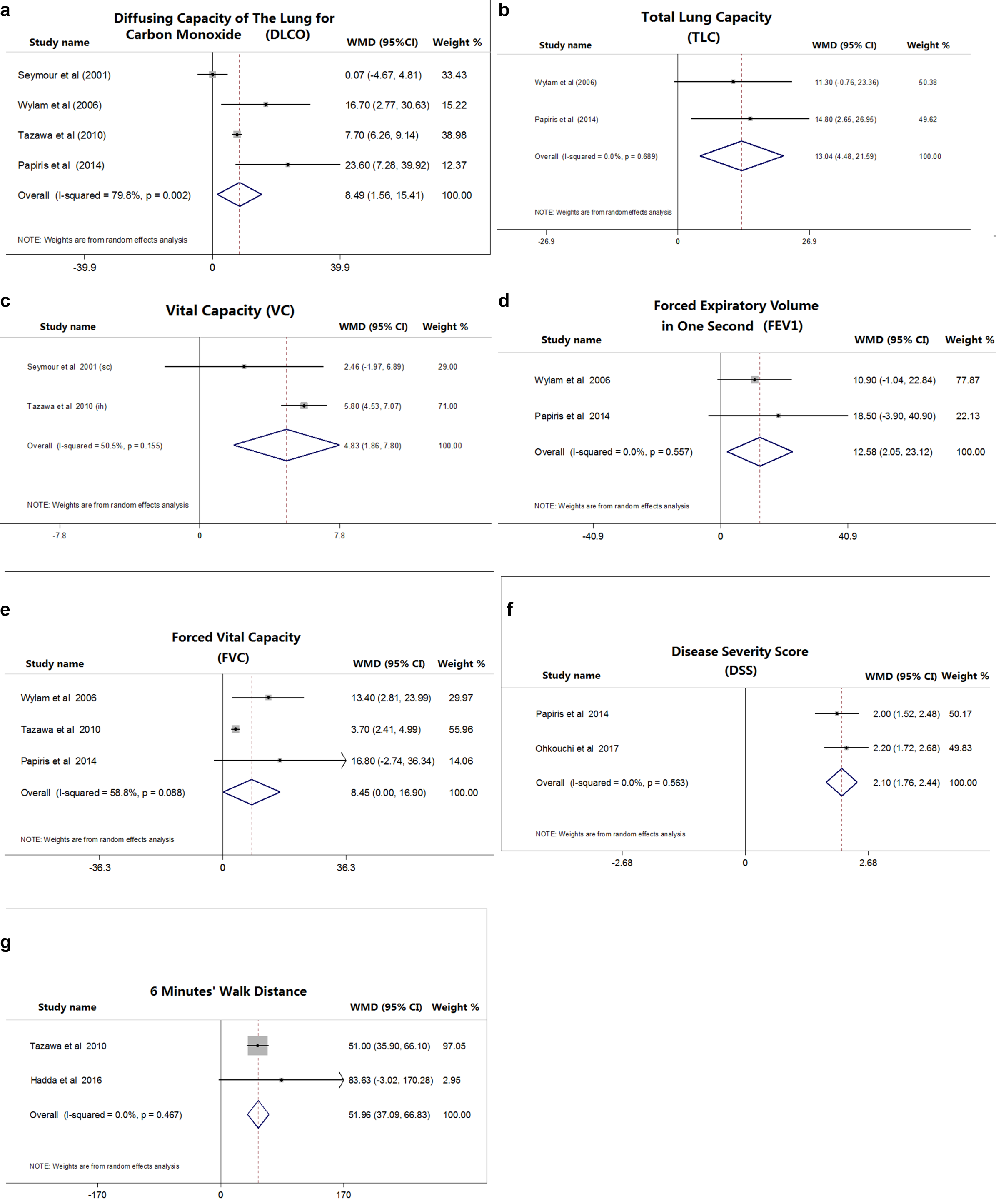


**Figure S8 (a).** Diffusing capacity of the lung for carbon monoxide (DLCO) improved by 8.49% (95%CI: 1.56-15.41, P=0.016) after GM-CSF therapy. **(b).** Total lung capacity (TLC) improved by 13.04% (95%CI: 4.48-21.59, P=0.003) after GM-CSF therapy. **(c).** Vital capacity (VC) improved by 4.83% (95%CI: 1.86-7.8, P=0.001) after GM-CSF therapy. **(d).** Forced expiratory volume in one second (FEV1) improved by 12.58% (95%CI: 2.05-23.12, P=0.019) after GM-CSF therapy. **(e).** Forced vital capacity (FVC) improved by 8.45% (95%CI: 0.003-16.9, P=0.05) after GM-CSF therapy. **(f).** Disease severity score (DSS) improved by 2.1 (95%CI: 1.76-2.44, P<0.001) after GM-CSF therapy. **(g).** 6 minutes’ walk distance (6MWD) improved by 51.96 (95%CI: 37.09-66.83, P<0.001) after GM-CSF therapy
